# Superiority of 1 h plasma glucose vs fasting plasma glucose, 2 h plasma glucose and HbA_1c_ for the diagnosis of type 2 diabetes

**DOI:** 10.1007/s00125-025-06632-y

**Published:** 2025-12-12

**Authors:** Yiying Wang, Jagannathan Ram, Cristina Bianchi, Teresa Vanessa Fiorentino, Sang Soo Kim, Jinmi Kim, Soree Ryang, Stefano Del Prato, Giorgio Sesti, Leontine Sandforth, Hubert Preissl, Reiner Jumpertz von Schwartzenberg, Norbert Stefan, Andreas Fritsche, Joon Ha, Andreas L. Birkenfeld, Michael Bergman

**Affiliations:** 1 Institute for Diabetes Research and Metabolic Diseases of the Helmholtz Center Munich at the University of Tübingen, Tübingen, Germany; 2 German Center for Diabetes Research (DZD), Neuherberg, Germany; 3 Internal Medicine IV, Division of Diabetology, Endocrinology and Nephrology, Eberhard-Karls University Tübingen, Tübingen, Germany; 4 Emory Global Diabetes Research Center, Woodruff Health Sciences Center and Emory University, Atlanta, GA USA; 5 Hubert Department of Global Health, Emory University, Atlanta, GA USA; 6 Department of Endocrine-Metabolic and Transplant Surgery and Medicine, University Hospital of Pisa, Pisa, Italy; 7 Department of Medical and Surgical Sciences, University “Magna Graecia” of Catanzaro, Catanzaro, Italy; 8 Department of Clinical and Molecular Medicine, Sapienza University of Rome, Rome, Italy; 9 Division of Endocrinology and Metabolism, Department of Internal Medicine, Pusan National University Hospital, Busan, Korea; 10 Biomedical Research Institute, Pusan National University Hospital, Busan, Korea; 11 Interdisciplinary Research Center “Health Science,” Sant’Anna School of Advanced Studies, Pisa, Italy; 12 Cluster of Excellence EXC2124 ‘Controlling Microbes to Fight Infections’ (CMFI), University of Tübingen, Tübingen, Germany; 13 M3 Research Center for Malignome, Metabolome and Microbiome, Faculty of Medicine, University of Tübingen, Tübingen, Germany; 14 Department of Mathematics, Howard University, Washington, DC USA; 15 Diabetes & Obesity Theme, School of Cardiovascular and Metabolic Medicine & Sciences, Faculty of Life Sciences & Medicine, Kings College London, London, UK; 16 NYU Grossman School of Medicine, Department of Medicine and of Population Health, Holman Division of Endocrinology and Metabolism, NYU Langone Diabetes Prevention Program, New York, NY USA

**Keywords:** 1 h plasma glucose, 2 h plasma glucose, Fasting plasma glucose, HbA_1c_, Intermediate hyperglycaemia, Meta-analysis, Type 2 diabetes diagnosis

## Abstract

**Aims/hypothesis:**

The IDF has proposed 1 h plasma glucose (1 h PG) as a diagnostic test for type 2 diabetes. This study evaluated the utility of 1 h PG in diagnosing type 2 diabetes, compared with fasting plasma glucose (FPG), 2 h plasma glucose (2 h PG), HbA_1c_ and the combination of HbA_1c_ plus FPG.

**Methods:**

Analyses were conducted using data from five independent cohorts: KoGES, CATAMERI, GENFIEV, PLIS (follow-up) and TULIP (follow-up). Type 2 diabetes was defined according to ADA criteria (FPG ≥7.0 mmol/l [≥126 mg/dl], 2 h PG ≥11.1 mmol/l [≥200 mg/dl] or HbA_1c_ ≥48 mmol/mol [≥6.5%]) or IDF criteria (1 h PG ≥11.6 mmol/l [≥209 mg/dl]). Area under of the receiver operating characteristic curves (AUC-ROCs) were used to assess the performance of 1 h PG relative to FPG and HbA_1c_, individually and in combination, for diagnosing diabetes. Random-effects meta-analyses were applied to pooled data to summarise the overall diagnostic accuracy across studies.

**Results:**

Cohort-specific analyses demonstrated consistently higher AUCs for 1 h PG in KoGES (AUC 0.96 vs 0.88; Δ 0.08; sensitivity 84.2 vs 77.0; specificity 98.6 vs 87.0), CATAMERI (AUC 0.98 vs 0.86; Δ 0.12; sensitivity 75.0 vs 69.4; specificity: 98.4 vs 78.9), GENFIEV (AUC 0.97 vs 0.89; Δ 0.08; sensitivity 89.5 vs 69.4; specificity 100.0 vs 88.3), PLIS follow-up (AUC 0.98 vs 0.76; Δ 0.22; sensitivity 94.9 vs 46.8; specificity 100.0 vs 92.3) and TULIP follow-up (AUC 0.98 vs 0.83; Δ 0.15; sensitivity 90.2 vs 90.2; specificity 100.0 vs 65.0) compared with FPG plus HbA_1c_ (all *p*<0.001). Meta-analysis of five cohorts (*N*=11,968) revealed superior diagnostic performance of 1 h PG compared with FPG plus HbA_1c_, with pooled AUCs (95% CI) of 0.97 (0.96, 0.98) vs 0.85 (0.82, 0.88).

**Conclusions/interpretation:**

These findings support the superior utility of the IDF-recommended 1 h PG vs FPG, 2 h PG, HbA_1c_ and FPG plus HbA_1c_ for diagnosing type 2 diabetes.

**Graphical Abstract:**

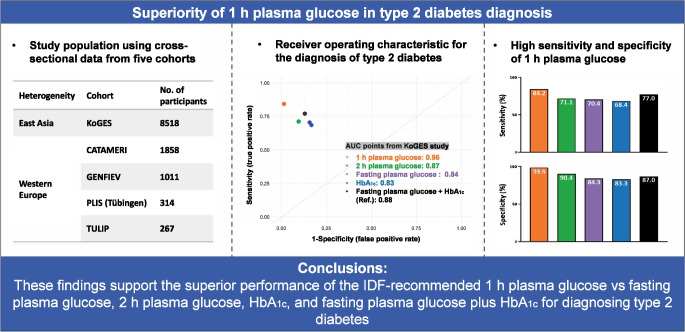

**Supplementary Information:**

The online version contains peer-reviewed but unedited supplementary material available at 10.1007/s00125-025-06632-y.


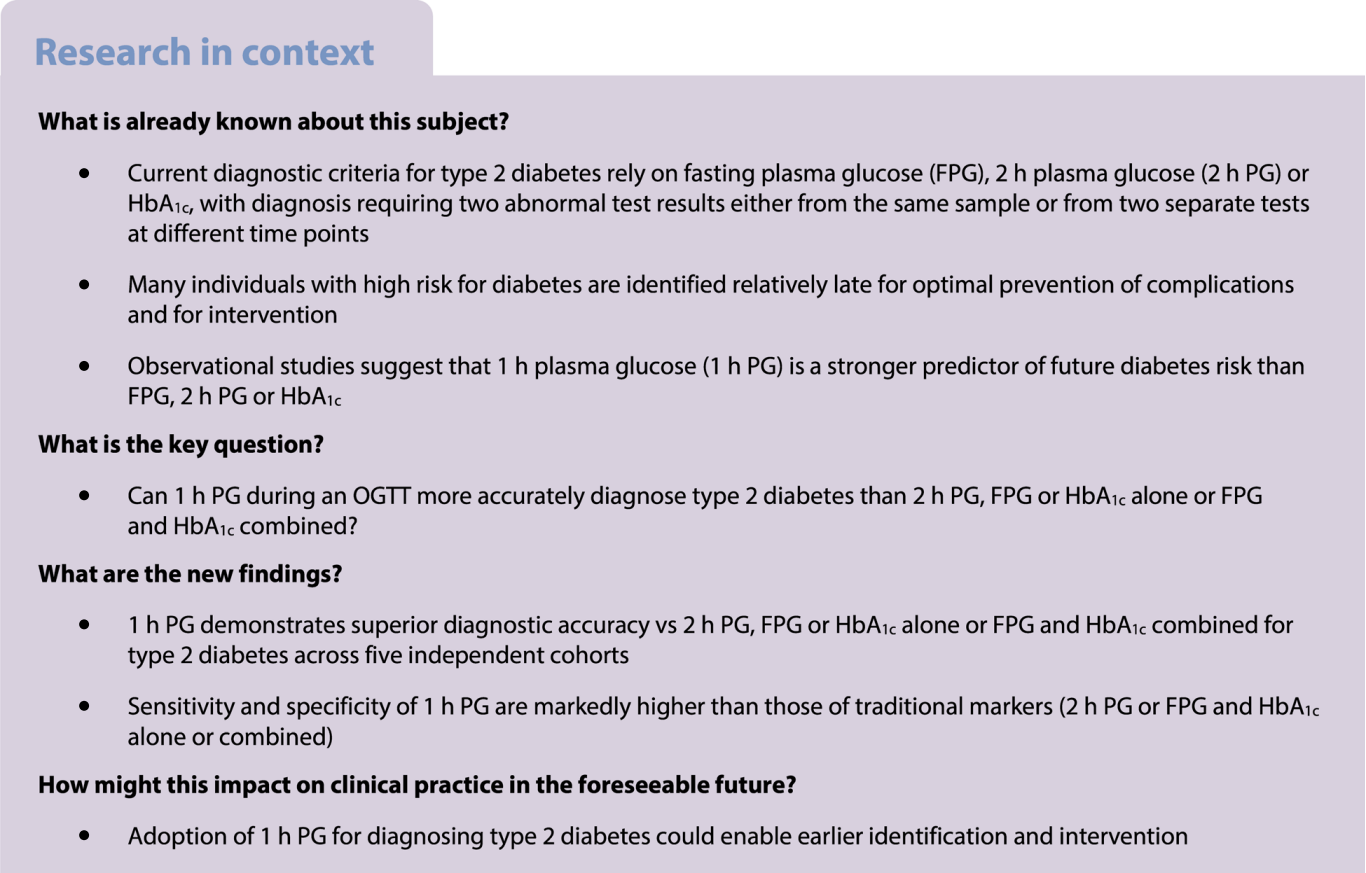


## Introduction

Current diagnostic measurements for type 2 diabetes have relied on fasting plasma glucose (FPG), 2 h plasma glucose (2 h PG) during an OGTT, or HbA_1c_ [1], but these often identify high-risk individuals late, when beta cell function is already impaired [2]. A combined use of FPG and HbA_1c_ has been endorsed by the ADA as a strategy to enhance diagnostic efficacy [3], supported by the ARIC study, a large prospective investigation of 13,346 participants followed for 25 years, showing that individuals with concurrent elevations in FPG (≥7.0 mmol/l [≥126 mg/dl]) and HbA_1c_ (≥48 mmol/mol [≥6.5%]) faced a 16-fold higher risk of developing diabetes [4, 5].

Notably, a 1 h PG cut-off of 11.6 mmol/l (209 mg/dl), identified by a meta-analysis of 15 studies involving 35,551 participants, demonstrated high sensitivity and specificity for detecting type 2 diabetes [6]. Furthermore, this threshold enabled diagnosis of type 2 diabetes approximately 1.4–1.6 years earlier than the traditional 2 h PG threshold of 11.1 mmol/l (200 mg/dl) [7, 8], which positions 1 h PG as a sensitive and practical tool for diagnosing type 2 diabetes.

In a Position Statement, the IDF endorsed the 1 h PG for diagnosing impaired glucose tolerance and type 2 diabetes [9]. Therefore, this study evaluated the utility of 1 h PG for diagnosing type 2 diabetes compared with 2 h PG or FPG and HbA_1c_ alone or in combination, in five studies involving Western European and East Asian populations in community-based settings and individuals at high risk.

## Methods

### Study population

This study analysed cross-sectional data from five independent cohorts. The Korean Genome and Epidemiology Study (KoGES) is a community-based cohort study investigating the incidence of and risk factors for non-communicable chronic diseases. It included a 2001–2002 baseline survey and follow-up every 2 years for 10 years, and enrolled individuals aged 40–69 years residing in the study area for ≥6 months; a 2 h OGTT was performed biennially [10]. The CATAMERI study is an observational investigation of White individuals with at least one cardiometabolic risk factor. Those without known diabetes underwent a 75 g OGTT [11]. The GENFIEV study is a multicentre Italian study recruiting at-risk individuals (e.g. diabetes family history, dyslipidaemia) via opportunistic screening to identify high-risk phenotypic/genotypic features, with a 75 g OGTT for non-diabetic individuals [12]. The Prediabetes Lifestyle Intervention Study (PLIS) is a randomised controlled multicentre trial across eight German university hospitals that evaluated the effect of an intensified vs regular lifestyle intervention in people with high- or low-risk prediabetes [13]. In this analysis, only participants from Tübingen University Hospital were included. The Tübingen Lifestyle Intervention Program (TULIP) is a German prospective intervention study of people at high risk for type 2 diabetes. It included participants identified by family history, BMI >27 kg/m^2^, impaired glucose tolerance, or history of gestational diabetes [14]. Only longitudinal follow-up data from PLIS and TULIP were assessed in this analysis.

### Definitions of type 2 diabetes

Type 2 diabetes was defined by meeting any ADA or IDF threshold: FPG ≥7.0 mmol/l (≥126 mg/dl); 2 h PG ≥11.1 mmol/l (≥200 mg/dl); HbA_1c_ ≥48 mmol/mol (≥6.5%); or 1 h PG ≥11.6 mmol/l (≥209 mg/dl) [1, 6, 9].

### Statistical analyses

The performance of 1 h PG in identifying diabetes was assessed relative to FPG, 2 h PG, HbA_1c_, and the combination of FPG and HbA_1c_ using the AUC for the receiver operating characteristic (ROC) curve and the sensitivity and specificity. ΔAUC was calculated as AUC_(single marker)_ − AUC_(FPG+ HbA1c)_. A meta-analysis of five cohorts was performed using random-effects models, with DerSimonian–Laird methods for AUC values and a bivariate random-effects model for sensitivity and specificity.

To assess the clinical impact, we calculated the number of diabetes cases that would have been missed by each individual diagnostic marker compared with 1 h PG, and further assessed whether adding HbA_1c_ or FPG to 1 h PG could improve diagnostic performance. In a sensitivity analysis, ROC analyses were repeated with diabetes being defined according to ADA criteria.

## Results

The characteristics of the study populations varied (Table 1), with mean ± SD ages ranging from 47.4 ± 11 to 58.5 ± 10 years, and the proportion of female participants ranging from 26.5% to 61.8%.

**Table 1:** ROC analysis of ADA diabetes criteria and 1 h post-load glucose for diagnosing type 2 diabetes

Diagnostic criterion	Participants	Age, years (mean ± SD)	Female (%)	AUC (95% CI)	△AUC	*p* value	Sensitivity	Specificity
KoGES	8518	51.6 ± 8	50.8					
FPG + HbA_1c_				0.88 (0.87, 0.89)	Ref.	Ref.	77.0	87.0
FPG				0.84 (0.82, 0.85)	−0.05	<0.001	70.4	84.3
1 h PG				0.96 (0.95, 0.97)	0.08	<0.001	84.2	98.6
2 h PG				0.87 (0.86, 0.88)	−0.01	0.171	71.1	90.4
HbA_1c_				0.83 (0.81, 0.84)	−0.06	<0.001	68.4	83.3
CATAMERI	1858	49.0 ± 14	49.8					
FPG + HbA_1c_				0.86 (0.84, 0.88)	Ref.	Ref.	69.4	78.9
FPG				0.83 (0.80, 0.85)	−0.03	0.010	57.4	76.1
1 h PG				0.98 (0.96, 0.99)	0.12	<0.001	75.0	98.4
2 h PG				0.91 (0.89, 0.93)	0.05	<0.001	59.1	90.2
HbA_1c_				0.80 (0.77, 0.83)	−0.06	<0.001	53.3	100.0
GENFIEV	1011	49.5 ± 11	26.5					
FPG + HbA_1c_				0.89 (0.86, 0.91)	Ref.	Ref.	69.4	88.3
FPG				0.86 (0.83–0.88)	−0.02	0.003	85.8	72.0
1 h PG				0.97 (0.96, 0.98)	0.08	<0.001	89.5	100.0
2 h PG				0.88 (0.85, 0.90)	−0.01	0.717	70.6	89.4
HbA_1c_				0.80 (0.77, 0.83)	−0.09	<0.001	69.4	88.3
PLIS (Tübingen)^a^	314	58.5 ± 10	60.5					
FPG + HbA_1c_				0.76 (0.70, 0.82)	Ref.	Ref.	46.8	92.3
FPG				0.75 (0.69, 0.82)	−0.01	0.707	57.0	81.7
1 h PG				0.98 (0.97, 1.00)	0.22	<0.001	94.9	100.0
2 h PG				0.79 (0.73, 0.85)	0.03	0.516	73.4	73.2
HbA_1c_				0.60 (0.60, 0.74)	−0.09	0.005	64.6	58.7
TULIP^a^	267	47.4 ± 11	61.8					
FPG + HbA_1c_				0.83 (0.77, 0.90)	Ref.	Ref.	90.2	65.0
FPG				0.83 (0.77, 0.89)	0.00	0.996	87.8	63.7
1 h PG				0.98 (0.96, 1.00)	0.15	<0.001	90.2	100.0
2 h PG				0.85 (0.78, 0.89)	0.03	0.573	82.9	71.7
HbA_1c_				0.68 (0.59, 0.78)	−0.15	<0.001	65.9	61.5
Meta five cohorts	11,968	51.1 ± 10	49.5					
FPG + HbA_1c_				0.85 (0.82, 0.88)	Ref.	Ref.	72.4	81.8
FPG				0.82 (0.79, 0.85)	−0.03	<0.001	72.1	81.3
1 h PG				0.97 (0.96, 0.98)	0.12	<0.001	88.9	98.5
2 h PG				0.86 (0.83, 0.89)	0.01	0.656	71.4	86.3
HbA_1c_				0.77 (0.70, 0.81)	−0.08	<0.001	64.4	75.4

^a^Values derived from the follow-up cross-sectional dataset

The reference approach combining FPG and HbA_1c_ yielded AUCs (95% CIs) of 0.88 (0.87, 0.89) in KoGES, 0.86 (0.84, 0.88) in CATAMERI, 0.89 (0.86, 0.91) in GENFIEV, 0.76 (0.70, 0.82) in PLIS follow-up and 0.83 (0.77, 0.90) in TULIP follow-up, with a meta-analysed AUC of 0.85 (0.82, 0.88). Sensitivity and specificity of this reference approach ranged from 46.8% to 90.2% and 65.0% to 92.3%, respectively, across cohorts.

When the diagnostic criteria were evaluated individually, FPG exhibited reduced discriminative ability compared with the reference, with a meta-analysed AUC of 0.82 (0.79, 0.85). The ΔAUC relative to the reference ranged from −0.05 to 0, while sensitivities ranged between 57.0% and 87.8% and specificities between 63.7% and 84.3%. The 2 h PG measurement also demonstrated comparable performance, with a meta-analysed AUC of 0.86 (95% CI 0.83, 0.89). The ΔAUC relative to the reference varied across studies (−0.01 to 0.05), with *p* values only indicating significant differences in the CATAMERI cohort (*p*<0.001). HbA_1c_ alone had the lowest AUCs among all markers across cohorts, ranging from 0.60 (95% CI 0.60,0.74) in PLIS to 0.83 (95% CI 0.81, 0.84) in KoGES. Compared with the reference, ΔAUCs ranged from −0.15 to −0.06, all statistically significant (*p*<0.01).

In contrast, the 1 h PG criterion consistently showed superior performance across all cohorts. The AUC for 1 h PG ranged from 0.96 (95% CI 0.95, 0.97) in KoGES to 0.98 (95% CI 0.97, 1.00) in PLIS, with a meta-analysed AUC of 0.97 (95% CI 0.96, 0.98), reflecting significant improvements over the reference (ΔAUC ranging from 0.08 to 0.22, *p*<0.001 in all cohorts).

Across cohorts, 1 h PG consistently identified more diabetes cases than FPG, HbA_1c_ or 2 h PG (electronic supplementary material [ESM] Table 1). Combining 1 h PG with HbA_1c_ or FPG yielded no significant clinically relevant improvement in AUC compared with 1 h PG alone (ESM Table 2). The optimal cut-off values derived from ROC analysis are presented in ESM Table 3. Using the ADA definition excluding 1 h PG, the 1 h PG maintained high diagnostic performance, with AUC values consistently superior to those for FPG and HbA_1c_ (ESM Table 4).

## Discussion

This analysis of five independent cohorts provides novel evidence that 1 h PG is superior to FPG, 2 h PG, HbA_1c_ or the combination of FPG and HbA_1c_ for diagnosing type 2 diabetes. The 1 h PG consistently demonstrated discriminative ability across all cohorts (AUCs 0.96–0.98) and significant improvements over the combination of FPG and HbA_1c_ (ΔAUC 0.08–0.22, *p*<0.001). Sensitivity and specificity of 1 h PG were high, reaching >90% sensitivity and specificity, whereas FPG, 2 h PG and HbA_1c_ exhibited moderate to lower AUCs (0.75–0.86, 0.79–0.91 and 0.60–0.83, respectively), with variable sensitivities (53.3–87.8%) and specificities (58.7–100%). These findings indicate that the 1 h PG outperforms both individual and combined traditional markers, providing significantly enhanced diagnostic performance and better-balanced sensitivity and specificity across diverse populations.

Selvin et al investigated the prognostic performance of combined FPG and HbA_1c_ measurements to confirm undiagnosed diabetes at an early stage [4]. While this approach showed high specificity (98.1%), it had moderate sensitivity (54.9%) at 5 years of follow-up. Importantly, as their study did not compare this strategy with OGTT-derived variables, the question of whether alternative markers, such as the 1 h PG, might provide superior diagnostic performance remained unresolved. Our findings address this gap by demonstrating that 1 h PG consistently outperforms FPG plus HbA_1c_, showing substantially higher sensitivities and specificities.

It is worth noting that the 2 h PG was found not to differ from the combination of FPG and HbA_1c_ (*p* for meta=0.656), strongly implying that a post-load glucose marker is important for improving the capacity to diagnose type 2 diabetes. The 1 h PG, as well as the FPG and 2 h PG, was found to be highly reproducible [15]. The 1 h PG offers a shorter, reproducible and more sensitive alternative to 2 h PG, can improve compliance with testing, and facilitates earlier intervention strategies [15]. Nevertheless, the 2 h PG remains valuable, particularly for prognostic assessment in individuals with impaired glucose tolerance or impaired fasting glucose at high risk of progression and adverse outcomes.

To the best of our knowledge, this is the first AUC-ROC analysis to elucidate the diagnostic performance of single and combined biomarkers for diagnosing type 2 diabetes. The key strength of this study lies in its analysis of large, heterogeneous cohorts from Western Europe and East Asia, enhancing the generalisability of findings. Furthermore, the consistent methodology across cohorts with varying study designs, combined with robust statistical comparisons, further strengthens the validity of our findings. Limitations of this analysis include the under-representation of certain populations, including Latin American, African, Pacific Islander, Southeast Asian and Middle Eastern cohorts and the enrichment of high-risk individuals defined by ADA criteria in PLIS/TULIP and GENFIEV. Nevertheless, evidence from South Korean [7] and Native American cohorts [8] supports the broader applicability of 1 h PG. Future research should assess the utility of 1 h PG in gestational diabetes and validate its performance in diverse youth populations.

In summary, this study reinforces the use of the 1 h PG as a criterion for diagnosing type 2 diabetes. Its superior diagnostic power and practical advantages over traditional markers positions the 1 h PG as a pivotal tool for the early diagnosis of type 2 diabetes.

## Supplementary Information

Below is the link to the electronic supplementary material.

Supplementary file1 (PDF 262 KB)

## Data Availability

The data analysed during the current study are not publicly available due to national data protection laws but are available from the corresponding author upon reasonable request.

## Abbreviations

1 h PG: 1 h plasma glucose
2 h PG: 2 h plasma glucose
FPG: Fasting plasma glucose
KoGES: Korean Genome and Epidemiology Study
PLIS: Prediabetes Lifestyle Intervention Study
ROC: Receiver operating characteristic
TULIP: Tübingen Lifestyle Intervention Program
