# Supplementary material for: Superiority of 1 h plasma glucose vs fasting plasma glucose, 2 h plasma glucose and HbA1c for the diagnosis of type 2 diabetes
Source: Diabetologia. 2025 Dec 12;69(4):911–6. doi: 10.1007/s00125-025-06632-y (PMC12957105; doi:10.1007/s00125-025-06632-y)
Supplement: Supplementary file 1 — Supplementary file1 (PDF 262 KB) [file 125_2025_6632_MOESM1_ESM.pdf]

Superiority of the 1 h plasma glucose vs fasting plasma glucose, 2 h plasma glucose and HbA_{1c} for the diagnosis of type 2 diabetes

Electronic Supplementary Material

ESM Table 1. The number of missed diabetes cases defined by individual diagnostic markers	……2
ESM Table 2. Receiver operating characteristic analysis of combining 1 h PG with HbA_{1c} or FPG for diagnosing type 2 diabetes	……3
ESM Table 3. Cutoff values from receiver operating characteristic analysis	……4
ESM Table 4. Receiver operating characteristic sensitivity analysis of ADA diabetes criteria for diagnosing type 2 diabetes.	……5

ESM Table 1. The number of missed diabetes cases defined by individual diagnostic markers

Study	KoGES (n=8,518)	CATAMERI (n=1,858)	GENFIEV (n=1,011)	PLIS (n=314)	TULIP (n=267)
**Diabetes cases, n**	1,120	386	275	79	56
**Diabetes cases defined by individual diagnostic markers (Missed cases), n**					
**FPG**	159 (961)	42 (344)	45 (230)	4 (75)	0 (56)
**1 h PG**	917 (203)	323 (63)	246 (29)	75 (4)	46 (10)
**2 h PG**	474 (646)	191 (195)	116 (159)	10 (69)	2 (54)
**HbA_{1c}**	439 (681)	88 (298)	75 (200)	4 (75)	9 (47)

ESM Table 2. Receiver operating characteristic analysis of combining 1 h PG with HbA_{1c} or FPG for diagnosing type 2 diabetes

Study	KoGES					CATAMERI					GENFIEV				
**Test Type**	**AUC (95% CI)**	**△ AUC**	**p-value**	**Sensitivity**	**Specificity**	**AUC (95% CI)**	**△ AUC**	**p-value**	**Sensitivity**	**Specificity**	**AUC (95% CI)**	**△ AUC**	**p-value**	**Sensitivity**	**Specificity**
**1 h PG**	0.96 (0.95-0.97)	Ref.	Ref.	84.2	98.6	0.98 (0.97-0.98)	Ref.	Ref.	86.3	99.5	0.97 (0.96-0.98)	Ref.	Ref.	89.5	100.0
**1 h PG + HbA_{1c}**	0.97 (0.97-0.98)	0.016	<0.001	90.9	94.8	0.98 (0.97-0.99)	0.004	0.130	94.8	92.2	0.97 (0.96-0.98)	0.004	0.118	90.2	97.8
**1 h PG + FPG**	0.97 (0.96-0.97)	0.009	<0.001	88.7	95.4	0.96 (0.96-0.97)	-0.011	0.001	88.6	91.9	0.97 (0.96-0.98)	0.002	0.334	91.6	96.3

Study	PLIS (Tübingen)					TULIP					Meta five cohorts				
**Test Type**	**AUC (95% CI)**	**△ AUC**	**p-value**	**Sensitivity**	**Specificity**	**AUC (95% CI)**	**△ AUC**	**p-value**	**Sensitivity**	**Specificity**	**AUC (95% CI)**	**△ AUC**	**p-value**	**Sensitivity**	**Specificity**
**1 h PG**	0.98 (0.96-1.00)	Ref.	Ref.	94.9	100.0	0.98 (0.96-1.00)	Ref.	Ref.	90.2	97.6	0.97 (0.96-0.98)	Ref.	Ref.	91.9	98.8
**1 h PG + HbA_{1c}**	0.98 (0.98-1.00)	0.002	0.206	93.7	98.7	0.99 (0.98-1.00)	0.006	0.829	95.1	94.7	0.98 (0.97-0.99)	0.004	0.255	93.9	96.2
**1 h PG + FPG**	0.98 (0.98-1.00)	0.001	0.192	96.2	98.3	0.98 (0.97-1.00)	0.001	0.744	92.7	96.7	0.97 (0.96-0.98)	-0.002	0.530	94.5	95.5

ESM Table 3. Cutoff values from receiver operating characteristic analysis

Study	KoGES	CATAMERI	GENFIEV	PLIS	TULIP
FPG (mg/dl)	91	99	97	105	95
1 h PG (mg/dl)	204	205	208	208	209
2 h PG (mg/dl)	152	154	161	133	127
HbA_{1c} (%)	5.9	5.7	5.8	5.9	5.7

ESM Table 4. Receiver operating characteristic sensitivity analysis of ADA diabetes criteria for diagnosing type 2 diabetes.

Study	KoGES					CATAMERI					GENFIEV				
**Test Type**	**AUC (95% CI)**	**△ AUC**	**p-value**	**Sensitivity**	**Specificity**	**AUC (95% CI)**	**△ AUC**	**p-value**	**Sensitivity**	**Specificity**	**AUC (95% CI)**	**△ AUC**	**p-value**	**Sensitivity**	**Specificity**
**FPG + HbA_{1c}**	0.94 (0.93-0.96)	Ref.	Ref.	84.8	94.7	0.90 (0.89-0.92)	Ref.	Ref.	84.1	82.4	0.92 (0.90-0.94)	Ref.	Ref.	82.0	89.8
**FPG**	0.88 (0.86-0.90)	-0.062	<0.001	74.8	89.2	0.85 (0.81-0.86)	-0.053	<0.001	77.0	77.9	0.87 (0.85-0.90)	-0.041	0.001	86.5	71.8
**1 h PG**	0.91 (0.89-0.92)	-0.035	<0.001	81.2	88.4	0.92 (0.90-0.93)	0.013	0.348	87.6	82.1	0.90 (0.88-0.92)	-0.019	0.019	85.8	81.8
**2 h PG**	0.92 (0.91-0.94)	-0.020	0.054	79.9	95.1	0.97 (0.96-0.98)	0.071	<0.001	89.4	96.0	0.93 (0.91-0.95)	0.011	0.626	77.4	99.1
**HbA_{1c}**	0.90 (0.89-0.92)	-0.038	<0.001	78.0	92.9	0.87 (0.85-0.88)	-0.036	0.002	81.0	76.2	0.85 (0.82-0.87)	-0.068	<0.001	74.0	84.0

Study	PLIS (Tübingen)					TULIP					Meta five cohorts				
**Test Type**	**AUC (95% CI)**	**△ AUC**	**p-value**	**Sensitivity**	**Specificity**	**AUC (95% CI)**	**△ AUC**	**p-value**	**Sensitivity**	**Specificity**	**AUC (95% CI)**	**△ AUC**	**p-value**	**Sensitivity**	**Specificity**
**FPG + HbA_{1c}**	0.85 (0.72-0.97)	Ref.	Ref.	81.2	95.0	0.90 (0.78-1.00)	Ref.	Ref.	81.8	98.8	0.94 (0.91, 0.97)	Ref.	Ref.	84.3	92.7
**FPG**	0.82 (0.66-0.98)	-0.037	0.145	68.8	93.0	0.89 (0.82-0.97)	-0.009	0.891	81.8	88.7	0.86 (0.85, 0.88)	-0.074	<0.001	80.4	80.2
**1 h PG**	0.84 (0.72-0.97)	-0.013	0.904	75.1	86.6	0.87 (0.79-0.96)	-0.031	0.726	100.0	61.7	0.90 (0.89, 0.92)	-0.034	<0.001	87.7	79.1
**2 h PG**	0.83 (0.68-0.98)	-0.024	0.84	68.8	100.0	0.85 (0.73-0.96)	-0.054	0.596	63.6	93.4	0.91 (0.87, 0.95)	-0.026	0.214	80.9	92.6
**HbA_{1c}**	0.86 (0.75-0.96)	0.003	0.937	81.2	81.2	0.89 (0.71-1.00)	-0.023	0.334	81.8	100.0	0.88 (0.85, 0.90)	-0.062	<0.001	80.7	86.2
